# Integration of Phenotype and Hormone Data during Adventitious Rooting in Carnation (*Dianthus caryophyllus* L.) Stem Cuttings

**DOI:** 10.3390/plants8070226

**Published:** 2019-07-15

**Authors:** María Salud Justamante, José Ramón Acosta-Motos, Antonio Cano, Joan Villanova, Virginia Birlanga, Alfonso Albacete, Emilio Á. Cano, Manuel Acosta, José Manuel Pérez-Pérez

**Affiliations:** 1 Instituto de Bioingeniería, Universidad Miguel Hernández, 03202 Elche, Spain; 2 Universidad Católica San Antonio de Murcia, Campus de los Jerónimos, 30107 Guadalupe, Spain; 3 CEBAS-CSIC, Campus Universitario de Espinardo, 30100 Murcia, Spain; 4 Departamento de Biología Vegetal, Universidad de Murcia, 30100 Murcia, Spain; 5 Dümmen Orange, 30890 Puerto Lumbreras, Spain

**Keywords:** vegetative plant propagation, hormone profiling, root architectural traits, stress-related hormones, ACC, IAA-Asp, water content, shoot growth

## Abstract

The rooting of stem cuttings is a highly efficient procedure for the vegetative propagation of ornamental plants. In cultivated carnations, an increased auxin level in the stem cutting base produced by active auxin transport from the leaves triggers adventitious root (AR) formation from the cambium. To provide additional insight into the physiological and genetic basis of this complex trait, we studied AR formation in a collection of 159 F_1_ lines derived from a cross between two hybrid cultivars (*2003 R 8* and *2101-02 MFR*) showing contrasting rooting performances. In three different experiments, time-series for several stem and root architectural traits were quantified in detail in a subset of these double-cross hybrid lines displaying extreme rooting phenotypes and their parental genotypes. Our results indicate that the water content and area of the AR system directly contributed to the shoot water content and shoot growth. Moreover, morphometric data and rooting quality parameters were found to be associated with some stress-related metabolites such as 1-aminocyclopropane-1-carboxylic acid (ACC), the ethylene precursor, and the conjugated auxin indol-3-acetic acid-aspartic acid (IAA-Asp).

## 1. Introduction

In contrast to most root tissues, adventitious roots (ARs) arise from non-root cells in response to some abiotic stresses or after wounding [1,2]. AR formation in stem cuttings is required for the vegetative propagation of many horticultural and forestry plant species and depends on a large set of exogenous and endogenous factors [3].

Carnation (*Dianthus caryophyllus* L.) plants are vegetatively propagated from terminal stem cuttings that undergo adventitious rooting, a process which has a strong genetic dependency and which leads to production losses in certain commercial cultivars [4]. As a first step towards the identification of the molecular pathways involved in this process, a quantitative description of rooting performance was obtained from ten carnation cultivars displaying extreme and contrasting phenotypic values selected from a wide collection of commercial lines [5]. The bad rooting cultivars were characterized by one or several of the following features: a delay in AR initiation, a reduced number of AR primordia, or a slow elongation rate of these ARs. In addition, genome-wide gene expression profiling and functional changes occurring in the stem cutting base during the early stages of adventitious rooting were further analyzed in two of these cultivars, *2003 R 8* and *2101-02 MFR*, which were selected because of their contrasting rooting performances [6]. Differences in rooting ability were caused by delayed activation of formative divisions from cambial-derived cells in the bad rooting cultivar (*2003 R 8*), a phenotype that could be rescued by exogenous auxin treatment [6]. Additional studies confirmed that the differential regulation of endogenous auxin homeostasis between these two carnation cultivars limited active auxin accumulation in formative cambial cells [7]. Despite the high auxin biosynthesis and transport rate in *2003 R 8*, the high expression of *GRETCHEN HAGEN3* (*GH3*) genes in the stem cutting base of this cultivar enhanced the conjugation of the active auxin indol-3-acetic acid (IAA) to amino acids, such as aspartic acid, which directly contributed to quick turnover of active auxin in the cambium [7].

To better understand the genetic basis of this complex trait, we studied AR formation in a collection of 33 selfed lines (from the *2101-02 MFR* cultivar) and 126 outcrossing lines from a cross between the *2003 R 8* and *2101-02 MFR* hybrid cultivars. Several morphological and physiological traits were quantified during rooting over different growing seasons in eight of these double-cross hybrid lines displaying extreme adventitious rooting phenotypes and their parental lines. Our understanding of the physiological and molecular events leading to the complex developmental response of AR formation in carnation stem cuttings will allow us to establish a marker-assisted selection approach to select for enhanced adventitious rooting traits during breeding of this economically important ornamental species.

## 2. Results

### 2.1. Phenotype Characterization of Parental Lines and the F_1_ Population Under Study

#### 2.1.1. Morphological Variation in *2003 R 8* and *2101-02 MFR* During Rooting

**Figure 1:**
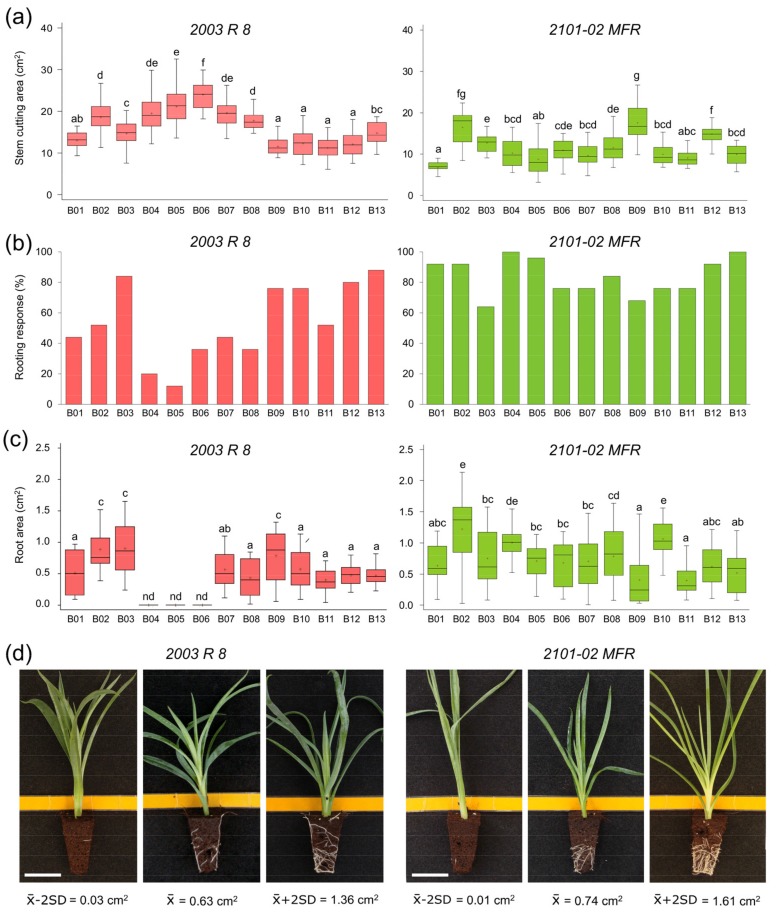
Quantitative description of the *2003 R 8* and *2101-02 MFR* parental lines among different batches. (**a**) The stem cutting area (cm^2^) at 13 days after planting (dap), (**b**) rooting response (%) and (**c**) root area (cm^2^) at 20 dap. The letters indicate significant differences between batches (Least Significant Difference [LSD]; *p*-value < 0.05). (**d**) Representative images of the phenotypic distribution of the root area (cm^2^) at 20 dap in *2003 R 8* (left) and *2101-02 MFR* (right). Scale bar: 25 mm.

Cold-stored stem cuttings periodically collected between April and June from mature mother plants of *2003 R 8* and *2101-02 MFR* lines were sequentially planted (sowings B01 to B13) on a demarcated greenhouse plot between June and October (see Materials and Methods). We found a significant variation in the stem cutting size, estimated by the SA_13 trait, between sowings in both parental lines (Figure 1a and Supplementary Materials Figure S1). Indeed, *2003 R 8* stem cuttings in several consecutive sowings (B04 to B07) displayed higher SA_13 values than the average (16.30 ± 5.01 cm^2^, n = 260) due to an increase in the average leaf width of those samples. On the other hand, the SA_13 values of *2101-02 MFR* were the lowest in B01 (7.74 ± 2.65 cm^2^) and the highest in B09 (17.60 ± 4.52 cm^2^), relative to the average SA_13 value throughout all sowings (11.5 ± 4.2 cm^2^, n = 260). These results confirmed the morphological heterogeneity in *2003 R 8* and *2101-02 MFR* stem cuttings during the production process, which is known to be dependent on environmental and physiological factors at the production plot [8].

The average values of the rooting response at 20 days after planting (dap) in *2003 R 8* and *2101-02 MFR* were 53.8 ± 24.9% and 84.0 ± 12.1%, respectively, which is consistent with previous findings [5,8]. The rooting response at 20 dap (RR_20) in *2003 R 8* stem cuttings was highly heterogeneous among different sowings (Figure 1b, left panel), and the lowest RR_20 values (<20%) corresponded to B04 and B05. Conversely, *2101-02 MFR* displayed a uniform rooting response (>60%) throughout all sowings (Figure 1b, right panel). The average values of the root area at 20 dap (RA_20) are a good predictor of the adventitious rooting performance, allowing a direct comparison between genotypes and experimental conditions [5,8]. Quantification of the adventitious rooting of the *2003 R 8* stem cuttings in B04 to B06 sowings was unsuccessful (Figure 1c, left panel), due to their low rooting responses (see above). These sowings overlapped with the warmest summer period (between 8 July and 13 August), which was characterized by the highest air temperature (~37 °C) registered inside the greenhouse plot during the experiment. Hence, we discarded the *2003 R 8* samples grown in the B04 to B06 sowings. The adventitious rooting performance in *2101-02 MFR*, estimated by RA_20, ranged three-fold, between 0.40 ± 0.24 cm^2^ in B11 and 1.22 ± 0.55 cm^2^ in B02, with an average value of 0.74 ± 0.43 cm^2^ (n = 250; Figure 1c, right panel). As described previously [6], the bad rooting behavior of *2003 R 8* was mostly caused by a delay in AR initiation (low RR_20 values), which contributed to the significant differences (Least Significant Difference [LSD]; *p*-value < 0.05) in RA_20 between *2003 R 8* (0.63 ± 0.36 cm^2^, n = 146; Figure 1d, left panel) and *2101-02 MFR* (0.74 ± 0.43 cm^2^, n = 250; Figure 1d, right panel).

#### 2.1.2. Morphological Variation of the Studied F_1_ Population

**Figure 2:**
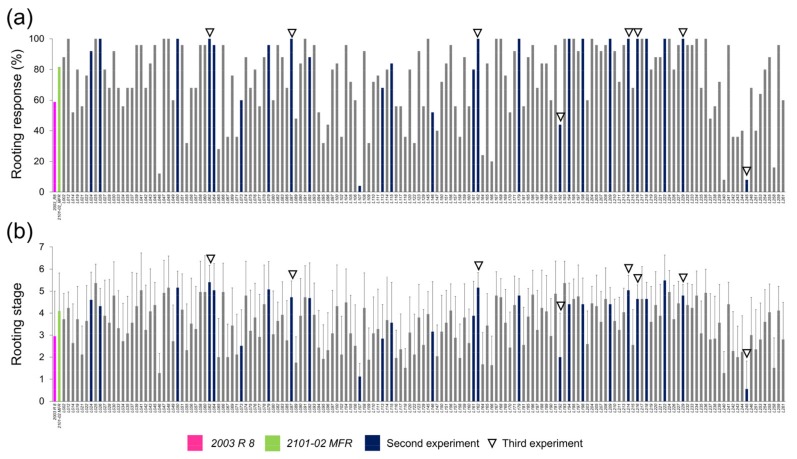
Quantitative description of some rooting parameters of the F_1_ lines derived from a cross between *2003 R 8* and *2101-02 MFR*. (**a**) Rooting response (%) and (**b**) rooting stage of the studied lines in the first experiment (n = 159) at 20 dap. The blue bars indicate the lines selected for the second experiment (n = 26), and the arrowheads indicate the lines selected for the third experiment (n = 8).

We measured six shoot traits and seven root traits (Table 1) that significantly changed during the rooting experiment (13 and 20 dap) in a population of 159 F_1_ lines obtained from a cross between *2003 R 8* and *2101-02 MFR* plants (see Materials and Methods). The average rooting response value in the studied F_1_ population at 20 dap (RR_20) was 74.6 ± 24.6%. The lowest rooting response values were found in *L107* (4%), *L240* (8%), and *L245* (8%), whereas twenty-nine F_1_ lines displayed full rooting (100%; Figure 2a). We defined eight rooting stages (RS) representing the different adventitious rooting phenotypes observed in carnation stem cuttings [5]. The average RS value of the F_1_ population ranged from 0.56 ± 1.3 (n = 25) in *L245* to 5.48 ± 1.1 (n = 25) in *L22*2 (Figure 2b).

We then performed a principal component analysis (PCA) with the remaining quantitative traits (see Materials and Methods). We found highly significant and positive correlations for traits related to the root system size (Supplementary Materials Figure S2a). Three principal components (PCs) accounted for 97.3% of the variation among the studied samples (Supplementary Materials Figure S2b). PC1 explained 65.3% of the variance and was positively dependent on the root thickness (estimated by ARD_20) but was negatively affected by the root convex area (RCA_20) and root number (MaxNR_20). PC2 and PC3 accounted for 24.4% and 7.6% of the variance, respectively. PC2 was explained by the root area (RA_20) and ARD_20, while PC3 was explained positively by RCA_20 and negatively by MaxNR_20. To visualize the effects of PC1, PC2, and PC3 on the root system architecture, representative images are shown in Supplementary Materials Figure 2c, where the PC values varied by plus or minus two standard deviations (SDs) from the mean.

#### 2.1.3. Transgressive Phenotypes in the Studied F_1_ Population

Despite the *2101-02 MFR* ovaries being used as receptors for *2003 R 8* pollen, 33 F_1_ lines were annotated as selfings based on microsatellite data (E.Á. Cano, personal communication). The remaining 126 F_1_ lines were double-cross hybrids between *2003 R 8* and *2101-02 MFR*. We next studied the phenotype distribution of selected rooting traits (ARD_20, RA_20, and MaxNR_20) in the parental lines, as well as in selfing (n = 33 lines) and outcrossing (n = 126 lines) subpopulations.

**Figure 3:**
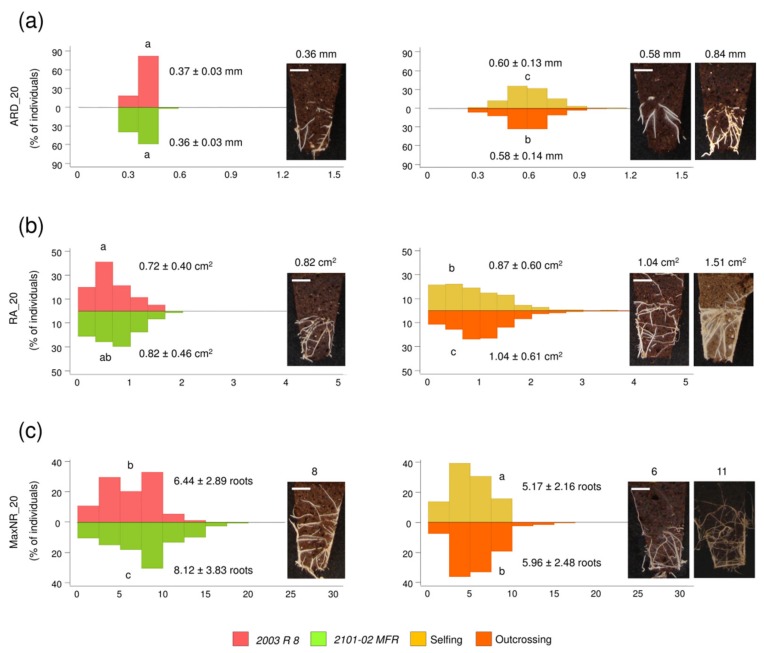
Examples of transgressive segregation observed in principal component analysis (PCA) relevant traits at 20 dap. (**a**) Average root width (ARD_20), (**b**) root area (RA_20), and (**c**) maximum number of roots (MaxNR_20) in the F_1_ population grouped by selfing (yellow) and outcrossing (orange) lines compared with *2003 R 8* (red) and *2101-02 MFR* (green) parental lines. Representative images of the average values of the best-rooting parental line *2101-02 MFR* (left) and average (medium) and maximum (right) values in the outcrossing subpopulation are shown. Letters indicate significant differences between the population groups (LSD; *p*-value < 0.05). Scale bar: 10 mm.

We previously determined that the root diameter (ARD_20) was negatively correlated with the adventitious rooting performance in a small population of carnation genotypes [5]. In this study though, we did not find significant variation in ARD_20 values between parental lines (Figure 3a, left panel), despite their contrasting rooting behaviors [6]. We found high levels of best-parent heterosis for this trait, with a ~60% increase in values in either selfing or outcrossing F_1_ subpopulations (Figure 3a, right panel and Supplementary Materials Table S1a). The highest ARD_20 values were found in the *L073* outcrossing line (0.84 ± 0.09 mm; n = 25), which corresponded to a 2.3-fold variation in the parental line and a 2.6-fold variation in the *L192* crossing line, with the lowest ARD_20 value (0.32 ± 0.07 mm; n = 25).

Regarding the AR area at 20 dap (RA_20), we found a slight variation between *2003 R 8* (0.72 ± 0.40 cm^2^; n = 175) and *2101-02 MFR* (0.82 ± 0.46 cm^2^; n = 250), as well as between selfing (0.87 ± 0.60, n = 825) and outcrossing (1.04 ± 0.61, n = 3150) subpopulations (Figure 3b). From these results, best-parent heterosis reached 0.3% and 22.0% in the selfing and outcrossing subpopulations, respectively (Supplementary Materials Table S1a). We observed a 1.3-fold variation in the maximum RA_20 value between *2101-02 MFR* and the outcrossing subpopulation (Figure 3b).

The root density in the soil plug was estimated by the MaxNR_20 trait, with statistically significant (LSD; *p*-value < 0.01) variation between *2003 R 8* (6.44 ± 2.89, n = 175) and *2101-02 MFR* (8.12 ± 3.83 cm^2^; n = 250). Interestingly, MaxNR_20 displayed negative heterosis both in selfing and outcrossing subpopulations (Figure 3c and Supplementary Materials Table S1a), despite some individual lines (e.g., *L079* and *L062*) displaying higher average values than the *2101-02 MFR* parental line.

### 2.2. Study of a Reduced Panel of Double-Cross F_1_ Hybrids

#### 2.2.1. Selection of Double-Cross F_1_ Hybrids and Heritability Estimation of the Studied Traits

Based on the results from the first experiment (see above), we selected 26 outcrossing lines displaying contrasting and severe rooting phenotypes (Figure 2a,b) to initiate the identification of the genetic determinants responsible for the rooting performance differences observed among carnation genotypes.

We found significant variation between the first and second experiments in the studied traits for most of the selected genotypes, which might indicate a strong environmental dependency of the studied traits that would make their further genetic dissection difficult. To reduce the environmental dependency on the studied traits, we discarded the F_1_ outcrossing lines with significant differences (LSD; *p*-value < 0.05) in RA_20 values and with unavailable plant material at the rooting station (E.Á. Cano, personal communication). As a result, eight outcrossing lines (Supplementary Materials Figure S3a) were selected.

Heritability (*H^2^*) results were estimated for these eight lines and compared to *H^2^*results obtained for the initial 26 outcrossing selected lines (see Materials and Methods). In all cases, the heritability values for the seven studied rooting traits increased when calculated for the eight selected lines. The heritability results had values ranging from 0.53 for the average root diameter at 20 dap (ARD_20) to 0.88 for the rooting stage at 20 dap (RS_20) (Supplementary Materials Figure S3b).

#### 2.2.2. Rooting Performance of Double-Cross F_1_ Hybrids

**Figure 4:**
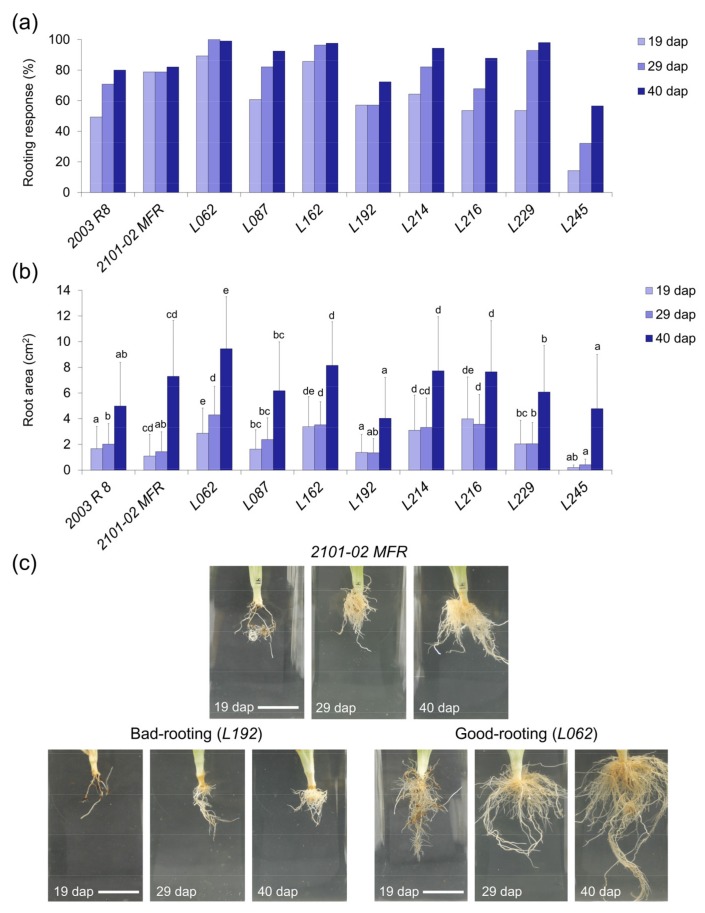
Adventitious rooting in the third experiment. (**a**) Rooting response measurements and (**b**) average root area (cm^2^) values in the studied lines at 19, 29, and 40 dap. Letters indicate significant differences between samples (LSD; *p*-value < 0.05). (**c**) Representative images of the *2101-02 MFR* parental line (up) and two lines with contrasting adventitious rooting phenotypes: the *L192* bad rooting line (left down) and the *L062* good rooting line (right down). Scale bar: 25 mm.

In the third experiment, we measured six shoot traits and nine root traits (Table 2) at planting times of 19, 29, and 40 dap (see Materials and Methods). Two of the studied F_1_ lines, *L062* and *L162*, displayed higher rooting response values at 19 dap (RR_19) than the good rooting parental line *2101-02 MFR*, while only *L245* showed lower RR_19 values than the bad rooting *2003 R 8* genotype (Figure 4a). Four other lines displayed higher (*L087*, *L214,* and *L229*) or lower (*L192*) RR values than the good rooting and bad rooting parental genotypes, respectively, at the other measured times (29 and 40 dap) (Figure 4a). The studied lines were clustered in five groups for RA_40, where *L062* was characterized by a significant RA increase compared with the good rooting parental line (Figure 4b,c). Despite *L162*, *L214,* and *L216* displaying significantly higher RA values at 29 dap than *2101-02 MFR*, their growth rate was maintained afterwards (40 dap). On the contrary, *L245* showed slightly reduced root growth throughout the experiment (Figure 4b,c). Taken together, and as previously described [5,8], the rooting performance of carnation genotypes appears to be influenced by a combinatorial effect of the rooting response and subsequent root growth, which was represented by the contrasting rooting behavior of *L192* and *L062* (Figure 4c)*,* with the former being moderately affected by the rooting stage but with reduced root growth as compared with the bad rooting *L245* line.

#### 2.2.3. Stem Cutting Ecophysiology of Double-Cross F_1_ Hybrids

**Figure 5:**
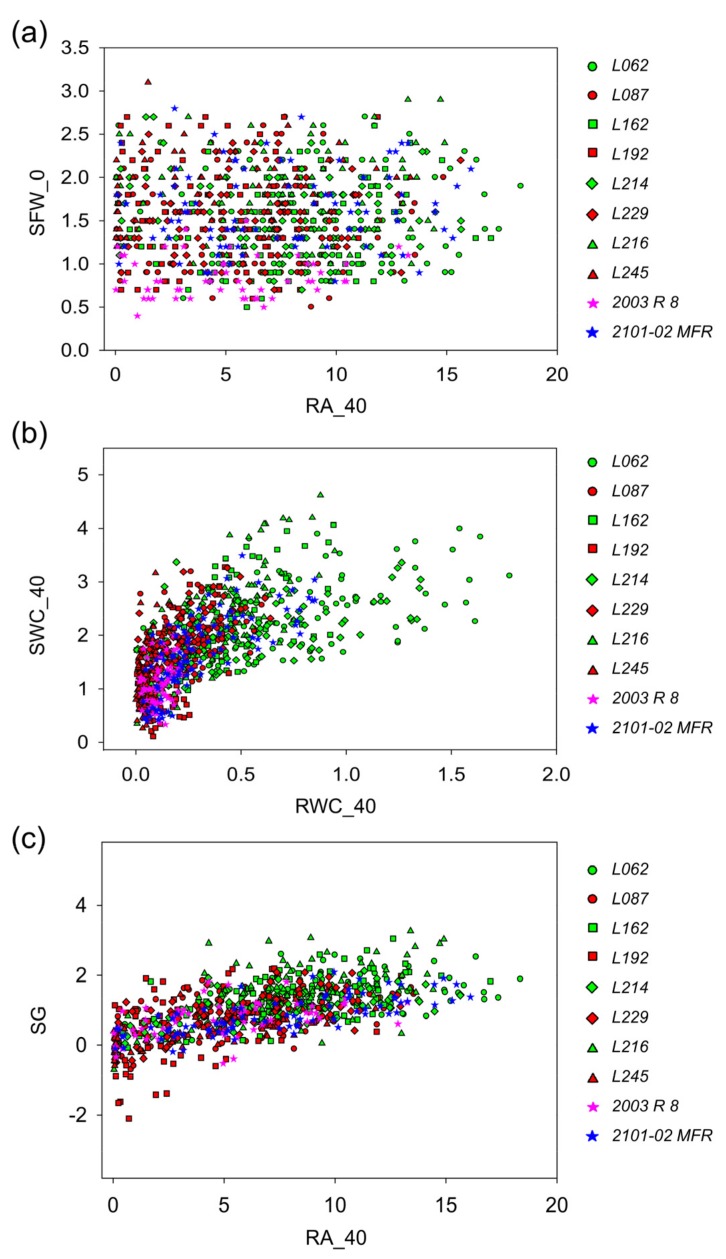
Parameter correlation observed in the third experiment for different physiological traits in the studied lines. Relationship between (**a**) the final root area (cm^2^) and the initial shoot fresh weight (g), (**b**) the root water content (g) and the shoot water content (g) and (**c**) the root area (cm^2^) and the final root growth. Each color and shape indicates one F_1_ studied line (good rooting lines in green and bad rooting lines in red), including parental lines (stars).

In the third experiment, we performed a series of analyses to determine whether physiological parameters of stem cuttings at planting time (SFW_0) correlated with the root area at the end of the experiment (RA_40). In addition, we determined whether the root area and its water content (RA and RWC) affected the shoot water content and its growth (SWC and SG) at the end of the experiment (Figure 5, Supplementary Materials Table S2). The correlation analysis between RA_40 and SFW_0 showed that the values of the root area at the end of the experiment could not be predicted from the initial shoot fresh weight (*p*-value > 0.05) and that there was no significant effect of the studied genotypes (Figure 5a and Supplementary Materials Table S2a). Conversely, positive and highly significant correlations (*p*-value < 0.001) indicated that the high water content of the root system (RWC) contributed to high water content values in the shoots (SWC) and to optimal shoot growth (SG) at the end of the experiment (Figure 5b,c, and Supplementary Materials Table S2b,c). In a similar way, additional correlation analyses between SWC and RA, SG and RWC, and RWC and RA were performed, and in most of the studied genotypes, the same highly-significant correlations were observed (*p*-value < 0.001) with large sample sizes in each genotype (Figures S4a–c, and Supplementary Materials Figures S2d–f).

Specifically, in the regression of SWC vs. RWC, genotypes with good rooting performances (green color) showed an average value in the RWC of 0.53 ± 0.35 g, which predicted an average value in the SWC of 2.22 ± 0.79 g. The genotypes with bad rooting performance (red color) presented comparatively lower average values than those of the good rooting ones, where 0.17 ± 0.14 g in the RWC predicted 1.62 ± 0.76 g in the SWC. Both parental lines *2101-02 MFR* (good rooting, blue color) and *2003 R 8* (bad rooting, pink color) showed lower values than selected lines with average values of 0.31 ± 0.22 and 0.12 ± 0.05 g in the RWC which equaled 1.76 ± 0.74 and 1.13 ± 0.40 g in the SWC, respectively (Figure 5b). Further, similar changes were also observed in the average RA values, as they could predict the values in SG (SG vs. RA_40 regression). Good rooting genotypes showed an average value in the RA_40 of 8.19 ± 3.90 cm^2^ that predicted an average value in SG of 1.25 ± 0.74 g. The genotypes with bad rooting performance presented lower average values than the good rooting ones. Namely, a RA_40 value of 5.30 ± 3.60 cm^2^ predicted a SG of 0.50 ± 0.95 g. The parental lines *2101-02 MFR* and *2003 R 8* showed lower values than the respective selected lines with average values of 7.38 ± 4.31 cm^2^ and 4.98 ± 3.39 cm^2^ in the RA_40, which predicted similar values in SG of 0.60 ± 0.70 g and 0.55 ± 0.61 g, respectively (Figure 5c).

#### 2.2.4. Capturing Variation in Morphometric, Ecophysiological, and Adventitious Rooting Traits in Carnation Stem Cuttings

**Figure 6:**
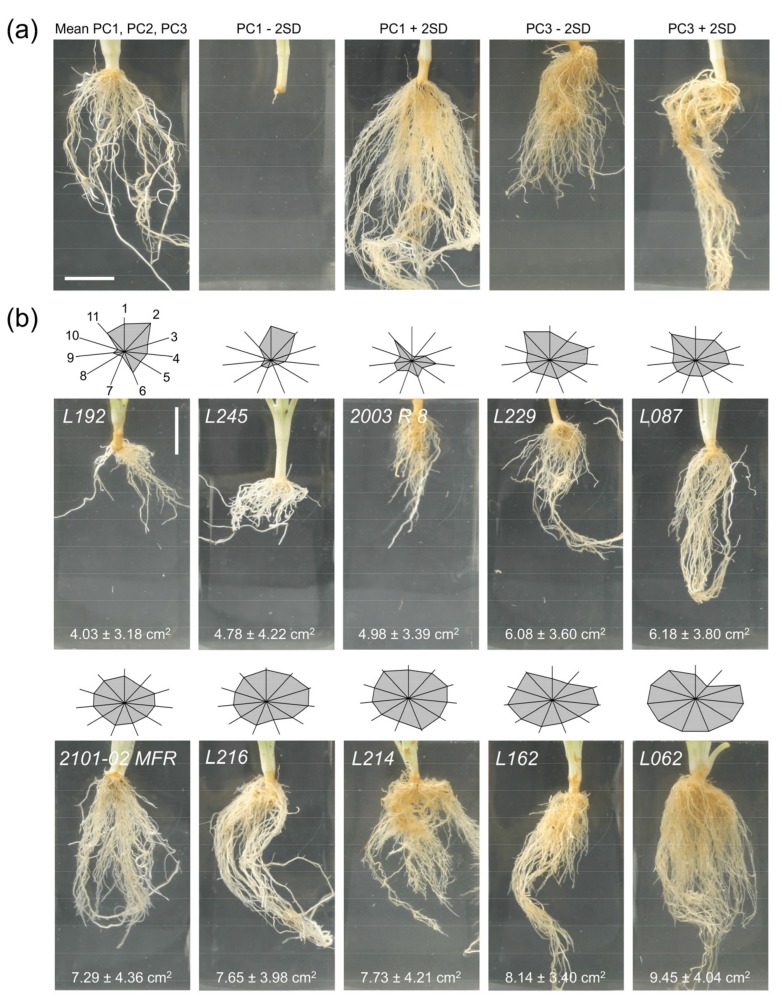
The double cross F_1_ hybrid lines with extreme rooting phenotypes studied in the third experiment. (**a**) Representative images corresponding to plus or minus two times the standard deviation (+2 SD and −2 SD) for PC1 and PC3. (**b**) The stars and rays graphs highlight the differences between genotypes. Each axis represents one parameter, and its intersection with the polygon sides indicates the relative magnitude for that parameter: (1) shoot fresh weight at planting time, (2) cutting length at planting time, (3) shoot water content, (4) shoot growth, (5) root water content, (6) average root width, (7) root area, (8) root convex area, (9) root depth, (10) root length, and (11) rooting response. Parameters 3 to 11 were measured at 40 dap. Representative images for the studied F_1_ lines are sorted by average root area values from the worst rooting line (*L192*) to the best rooting one (*L062*). Scale bar: 20 mm.

Through correlation analysis and PCA, we aimed to predict the rooting performance using a reduced number of the measured traits (see Materials and Methods). Three PCs accounted for 80.8% of the variation at 40 dap (Supplementary Materials Figure S5a). PC1 explained 48.9% of the variance and was strongly influenced by the root system size (e.g., RA_40, RL_40), while PC2 accounted for 21.7% of the variation and was positively influenced by shoot morphological and physiological parameters, such as SFW_0 and CL_0. PC3 explained 10.2% of the variation and was mostly influenced by the root diameter (ARD_40). Consistent with the high correlation found between most vegetative and rooting parameters (see above and Supplementary Materials Figure S5b), PC1 clearly clustered different vegetative and rooting traits. Figure 6a includes representative stem cutting images corresponding to plus or minus two times the standard deviation for PC1 and PC3. To help visualize the relative effect of each trait on the rooting performance, we built a star and rays graph for the double cross F_1_ hybrids studied, which were ordered based on their average RA_40 values (Figure 6b). In agreement with these results, *L192* and *L245* displayed lower rooting performances than their bad rooting parental line (*2003 R 8*), while *L162* and *L062* displayed higher rooting performances than their good rooting parental line (*2101-02 MFR*).

#### 2.2.5. Hormonal Profiling in the Stem Cutting Base During the Rooting of Double-Cross F_1_ Hybrids

**Figure 7:**
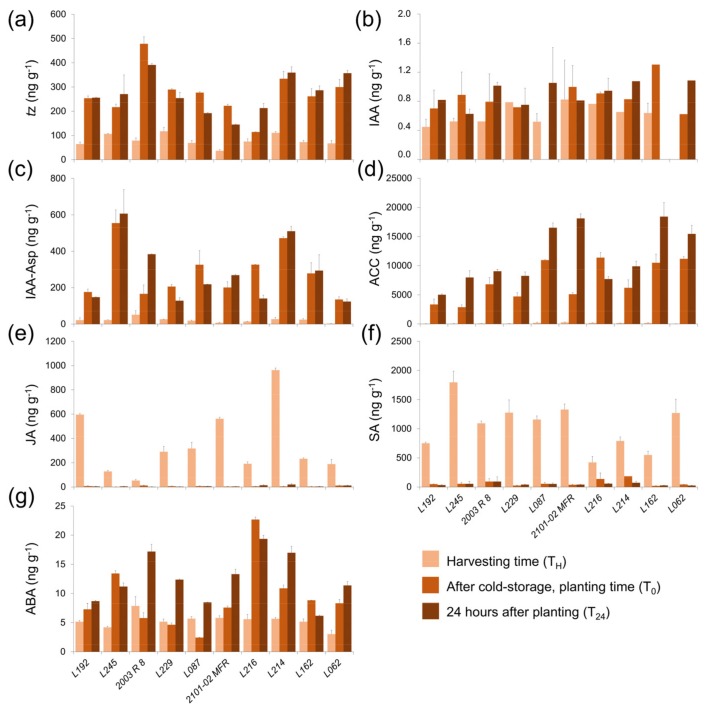
Hormonal profiling in selected lines for the third experiment. Bar charts at harvesting time (T_H_), at planting time (T_0_), and 24 hours after planting (T_24_) for (**a**) *trans*-zeatin (*t*Z), (**b**) indole-3-acetic acid (IAA), (**c**) indole-3-acetyl-aspartate (IAA-Asp), (**d**) 1-aminocyclopropane-1-carboxylic acid (ACC), (**e**) jasmonic acid (JA), (**f**) salicylic acid (SA), and (**g**) abscisic acid (ABA).

We measured endogenous cytokinin (CK; *trans*-zeatin and zeatin riboside) levels in the stem cutting base during rooting. We found lower *trans*-zeatin levels in freshly harvested stem cuttings in all studied genotypes and these significantly increased after cold storage (Figure 7a). Compared to steady state levels, the average *trans*-zeatin level increased from 2.0-fold in *L245* to 6.1-fold in *2003 R 8* after cold storage. The *trans*-zeatin level was not significantly increased in the studied genotypes 24 h after planting. Together, we did not find any trend between CK levels and rooting performance in the double-cross F_1_ hybrids studied (Figure 7a). However, we found a clear distinction between the *2003 R 8* and *2101-02 MFR* parental genotypes, due to *2003 R 8* stem cuttings having doubled or quadrupled *trans*-zeatin levels at harvest and at planting time, respectively, as compared to those in *2101-02 MFR*.

We found low indole-3-acetic acid (IAA) levels in the stem cutting base of double-cross F_1_ hybrids during the experiment, irrespective of their rooting performance (Figure 7b). Some inactive IAA conjugates, such as IAA-Asp, have previously been found at low levels at harvest time with a significant increase after cold storage [8]. In addition, the time course differences in IAA-Asp levels between the *2003 R 8* and *2101-02 MFR* lines might partially account for their contrasting rooting performances [7]. In agreement with our previous results, we found low IAA-Asp levels at harvest time and a significant increase after cold storage in all the studied genotypes (Figure 7c). Interestingly, contrasting IAA-Asp values were found in *L062* and *L245* throughout the experiment, which also corresponded to the best-rooting and worst-rooting double-cross F_1_ hybrids studied (see above).

The level of 1-aminocyclopropane-1-carboxylic acid (ACC) in the stem cutting base is an indirect estimate of the ethylene concentration [10]. Confirming our previous results [8], low ACC levels were found in the freshly harvested stem cuttings of the studied genotypes, whereas ACC levels significantly increased during cold storage and were higher in the *2101-02 MFR* genotype compared with *2003 R 8* at the moment of planting (6802.67 and 11381.51 ng g^-1^, respectively; Figure 7d). Interestingly, most bad rooting genotypes, such as *L192*, *L229,* and *L245*, displayed much lower ACC levels at planting time than those in the good rooting cultivars (*L062* or *L162*, Figure 7d), which suggests a positive correlation between rooting performance and endogenous ACC levels in the stem cutting base at planting time in this species. Unusually, the *L087* line displayed high ACC levels at planting time and a bad rooting performance (Figure 7d). Jasmonic acid (JA) has been recently connected to a positive role in wound-induced adventitious rooting in several species through crosstalk with auxin and ethylene signals [11,12,13]. Thus, the JA level at harvesting time might be considered a direct read-out of the wound signal due to its quick turnover in carnation stem cuttings [8]. We did not find any clear correlation between the JA level at harvesting time (Figure 7e) and the rooting performance in carnation double-cross F_1_ hybrids, as the highest JA-producers (*L192* and *L216*) displayed contrasting rooting phenotypes (Figure 6b). These results supported the hypothesis that the overall endogenous JA level is not critical for adventitious rooting in carnation stem cuttings. We also found significant variation in the endogenous SA level at harvesting time between the studied genotypes, which was reduced to the steady-state level during cold storage (Figure 7f). Despite this, we did not find any significant correlation between the endogenous SA level and the rooting performance; most of the bad rooting cultivars were characterized by a combination of low JA and high SA at harvest time, with the exception of the good rooting *L062* line. The highest ABA levels at harvest time were found in the *2003 R 8* parental line (7.86 ng g^-1^; Figure 7g). In many cases, the endogenous ABA levels significantly increased during cold storage, except in *2003 R 8*, *L087,* and *L229,* and the highest fold-increase was observed in *2101-02 MFR*, followed by the bad rooting line *L245* (Figure 7g). These results indicate that endogenous ABA levels during early rooting are not directly correlated with the rooting performance in the double-cross F_1_ hybrids studied, although they might contribute to stress adaptation during subsequent root growth.

## 3. Discussion

We previously reported that differences in the physiological status of *2003 R 8* and *2101-02 MFR* mother plants strongly contribute to successful adventitious rooting [8]. Our results confirm the morphological heterogeneity in stem cutting production and reveal high environmental dependence for *2003 R 8*, which also displayed a negative correlation between the leaf area at collection time and the rooting performance. Indeed, a large stem cutting area might directly contribute to significant water loss at planting time and before the newly produced adventitious root (AR) system is functional. A study performed in rice [14] identified the AR length as a useful marker for evaluating drought stress in different genotypes. Hence, carnation cultivars with a reduced stem cutting area and rapid AR production, such as *2101-02 MFR*, will handle water stress more efficiently than other genotypes.

Our F_1_ population initially consisted of 114 selfed lines (from the *2101-02 MFR* cultivar) and 148 double-cross hybrid (i.e., outcrossing) lines derived from a cross between the *2003 R 8* and *2101-02 MFR* hybrid cultivars (E.Á. Cano, personal communication). We performed quantitative phenotyping of stem cutting morphology in a subset of these lines, which were selected based on breeding performance during stem cutting production. As a result, only 28.9% of the selfed lines (in contrast to 85.1% of outcrossing lines) were studied, which was in agreement with previous results of inbreeding depression in this species [15,16]. Adventitious rooting in soil plugs was studied as described previously [5], and transgressive segregation was found in both F_1_ subpopulations for most of the studied traits. Interestingly, the AR area (RA_20) displayed high heterotic values in the outcrossing F_1_ subpopulation as compared with the good rooting *2101-02 MFR* parental line, which suggests an additive effect of favorable alleles from both parental lines on AR growth and development. In a recent study in cucumber, a large F_2_ population derived from two contrasting lines differing in the AR number during waterlogging stress was studied, and the genetic dissection of this quantitative trait has been initiated [17]. We also found high heterotic values for the AR diameter (ARD_20) in both F_1_ subpopulations that deserve further investigation. In contrast, previous experiments reported low levels of heterosis in the primary root diameter in maize during early development [18].

To initiate the genetic dissection of AR development in this species, in a second experiment, we evaluated several AR traits in 26 outcrossing lines displaying contrasting heterotic values. We discarded most of these lines for further experiments due to strong genotype × environment (G × E) interactions or unavailable plant material (E.Á. Cano, personal communication). We found higher heritability values on AR traits when considering only the selected lines that were environmentally stable. However, in some AR traits, heritability values were low (e.g., ARD_20), which might complicate subsequent genetic analyses. Some studies have initiated the genetic analysis of AR development in several species, such as teosinte [19], bean [20], rice [21], poplar [22], barley [23], and cucumber [17], with variable success. In the poplar study [22], despite moderate heritability for the AR number (*H^2^*  =  0.27–0.34), the integration of genetic, genomic, and transcriptomic information allowed the identification of two quantitative trait loci (QTL) for this trait. Their results suggest that the difference in the AR numbers of two contrastingly distinct species, *Populus deltoides* and *Populus trichocarpa*, is driven by the difference in the expression of two genes in the IAA biosynthesis pathway, possibly the poplar homologues of *SUPERROOT2* (*SUR2*) and *TRYPTOPHAN SYNTHASE ALPHA CHAIN* (*TSA1*).

In a third experiment, we studied several physiological and adventitious rooting traits in a selected population of double-cross F_1_ hybrids and their parental lines, *2003 R 8* and *2101-02 MFR*. For a complete visualization of their AR systems, we removed substrates at different times after planting (dap). At 40 dap, we identified three PCs associated with 80.8% of the variation. Traits related to the AR system size positively contributed to PC1 (48.9% of the observed variance), while shoot traits at planting time positively contributed to PC2 (21.7% of the variance). These results are in agreement with our previous observation that large stem cuttings displayed lower rooting performances [5], likely due to their higher drought stress sensitivity. Root system architecture traits, such as length, diameter, and area, determine root performance, enabling plants to acquire water and nutrients and thereby increase the replacement rate of their water loss [24]. Optimum root systems can support shoot growth and improve plant yield, since roots are the functional interface between plants and the soil [25]. An enlarged root system will penetrate into deeper layers of soil to acquire water and nutrients more efficiently [26]. Our study identified a key correlation between a large, functional root system (estimated by RA_40 and RWC) and proper water balance in the shoot, which is essential for shoot growth (SWC and SG, respectively). Interestingly, a significant amount of the observed variation was influenced by the root diameter, which greatly contributed to PC3 (10.2%). A recent study on wheat indicated that the root diameter plays an important role in phosphorus-deficiency tolerance, as it defines the volume of soil in contact with the roots [27]. In this and other species, the survival ability of individual roots increases with an increasing root diameter [28]. However, the effect of the root diameter on plant performance is controversial, as the increase in this trait has been shown to promote nutrient uptake due to increases in xylem and phloem tissues as the surface area increases [29], while seemingly relatively thinner roots might be more effective at absorbing soil phosphate. Therefore, an increased carnation AR root diameter might negatively contribute to limiting the nutrient uptake (i.e., phosphorus) during early AR development and thus restrict root growth. Our previous results with a limited number of carnation genotypes suggest that the root diameter is negatively correlated with the rooting performance [5]. Our current results show a low correlation between the AR diameter and AR system size traits. Consequently, it remains unclear whether the AR diameter increases or decreases the AR performance of carnation stem cuttings, and additional experiments will be required to determine whether the AR diameter should be included as a positive or negative trait during breeding.

In our study, the double-cross F_1_ hybrids *L192* and *L245* displayed poor rooting performances as compared with the bad rooting *2003 R 8* parental line. However, significant differences were found in the JA, SA, and IAA-Asp levels in the stem cutting bases of *L192* and *L245* during the experiment. *L245* presented a lower rooting response and more retarded root growth than *L192*, which was correlated with the high SA/JA proportion at harvest time and the high IAA-Asp level during rooting in the *L245* line. Low levels of exogenously-applied SA promoted AR formation in mung bean (*Vigna radiata*) hypocotyl cuttings, but an increased SA concentration inhibited this process [30]. As in most species [31], adventitious rooting is also strongly influenced by auxin homeostasis in carnations [7], and one intriguing possibility is that unknown SA–JA–IAA crosstalk might play a regulatory role in adventitious rooting. *L062* and *L162* displayed better rooting performance than the good rooting *2101-02 MFR* parental line. The ACC levels were quite high in these good rooting lines while their IAA-Asp levels were moderate. High levels of ACC after cold storage and during early rooting might reflect increased ethylene production after planting, which might contribute to enhanced adventitious rooting, as suggested previously [6]. In mung bean cuttings, exogenous ethylene application promoted AR formation, with a positive effect during AR initiation [32]. There is additional evidence of direct crosstalk between ethylene and auxin transport during AR formation [33,34], and further experiments are needed to elucidate if similar crosstalk is operating in carnation stem cuttings.

The rooting ability in carnation stem cuttings involves a multifactorial process that is regulated by different phytohormones, where endogenous auxin homeostasis and stress-related metabolites such as ethylene might play essential roles. Regulation of these hormonal pathways can establish the AR quality of each line and the improvement of some morphological and physiological traits involved in this process, as we have seen here. Further studies are necessary in order to improve the characterization of the studied cultivars and to determine the causative molecular basis. To this end, time series transcriptome analysis during AR formation in bulked samples from bad rooting (*2003 R 8*, *L192* and *L245*) and good rooting (*L062* and *L162*) F_1_ hybrids will allow the identification of differentially regulated genes with alternative alleles that could be used to map some of the QTLs involved in the observed differences in adventitious rooting quality traits.

## 4. Materials and Methods

### 4.1. Plant Material, Growing Conditions, and Sample Collection

Carnation cultivars *2003 R 8* and *2101-02 MFR* with contrasting rooting phenotypes were obtained from Dümmen Orange (http://www.dummenorange.com). The studied population consisted of 159 F_1_ lines derived from a cross between these parental cultivars, which included 33 selfed lines (from the *2101-02 MFR* cultivar) and 126 outcrossing lines. Mother plants from all of these lines and the two parental cultivars were grown in the same greenhouse and under environmental conditions at 37°34′50″N, 1°46′35″W and at an altitude of 395 m (Puerto Lumbreras, Murcia, Spain).

#### 4.1.1. First and Second Experiments

For each genotype, commercial quality stem cuttings were pinched from several mature (>1 year old) mother plants by skilled operators every 15 days from 7 April to 30 July 2014. Stem cuttings were immediately wrapped in plastic bags and stored in a cold chamber at 5 ± 2 °C and 60% relative humidity in complete darkness. For each experiment, 20–25 stem cuttings from each genotype were randomly selected and individually planted, as described previously [5].

The first experiment was divided into 10 batches (B01 to B10), and the stem cuttings of different lines were planted from 17 June to 9 September 2014. Twenty-six outcrossing F_1_ lines from the first experiment with contrasting rooting phenotypes were selected for the second experiment. This second experiment included three additional batches (B11 to B13), and stem cuttings were planted from 7 October to 29 October 2014. All stem cuttings were individually planted in moistened peat-perlite (90/10 *v/v*) substrate trays of truncated pyramid plugs (2.5 × 2.5 × 4.0 cm; 16 cm^3^), as indicated above. Water, fertilizer, and adequate phytosanitary treatments were periodically applied [5,35].

#### 4.1.2. Third Experiment

With the results obtained in the previous two experiments, eight F_1_ outcrossing lines with extreme rooting phenotypes were selected based on the rooting stage and root area average values at 20 dap. Carnation stem cuttings from these selected lines and the parental lines were pinched from several mature mother plants by skilled operators from 23 January to 19 April 2017. Stem cuttings were wrapped in plastic bags and stored in a dark and cold chamber at 5 ± 2 °C. 

A total of 106 cold-stored stem cuttings of each genotype were randomly selected and individually planted in moistened peat-perlite (50/50 *v/v*) substrate trays of truncated pyramid plugs (3.8 × 3.8 × 4.0 cm; 32 cm^3^). Stem cuttings were grown from 11 May to 20 June 2017 in a single batch (B14), on a gothic-arch greenhouse at 38°16’43´´ N, 0°41´15´´ W at an altitude of 96 m (Elche, Spain) under environmental conditions and with periodic sprinkler irrigation.

At planting time (0 dap), the stem cutting length and fresh weight were measured in each sample. At the end of the experiment (40 dap), fresh weights were separately measured for shoot and root tissue in each sample. For dry weight measurements, shoot and root tissues were dehydrated for three days in a stove at 80 °C (J.P. Selecta S.A., Barcelona, Spain) and weighed on a high precision scale (PCE Instruments, Spain).

For hormone analysis, three replicates of each genotype, each consisting of 12 (T_0_ and T_24_) or 25 (T_H_) stem cutting bases (about 4 mm long), were collected at three different times: (T_H_) when stem cuttings were collected from the mother plants, (T_0_) at planting time (0 dap), and (T_24_) 24 hours after planting (1 dap). Samples were immediately frozen in liquid nitrogen and stored at −80 °C.

### 4.2. Picture and Image Processing

#### 4.2.1. Image Collection

For the first and second experiments, stem cutting pictures were taken from one side of the soil plug at 13 dap (n = 20 stem cuttings) and 20 dap (n = 25 stem cuttings). Pictures were taken using a Canon 60D camera with a Canon EF-S 17-85 mm f/4-5.6 IS USM lens with a resolution of 5184 × 3466 pixels. 

Pictures of 28 randomly selected stem cuttings from the third experiment were taken from one side of the soil plug at 19 and 29 dap. The soil plug was carefully removed at 40 dap by washing it with high-pressure tap water, and the entire root system was imaged after transferring it to a water bath (n = 106 stem cuttings). Pictures were taken using a Canon EOS camera with a Canon EF-S 18-55 mm lens and a resolution of 4272 × 2848 pixels.

To minimize variations due to sunlight quality during the day, a portable photographic bench was used, and all the pictures were taken at the greenhouse between 11 a.m. and 1 p.m. These pictures were saved as an RGB color image in jpeg format. All original pictures are available upon request.

#### 4.2.2. Image Processing

In each picture, stem and root tissue were separated by using the Multiple Image Processor plug-in of ImageJ (https://imagej.net/) and batch-imported into GiA Roots software [9]. After scale calibration and grayscale conversion (RGB to gray simple conversion option), stem cutting images were segmented using the Global Thresholding method, and soil plugs and root images were segmented using the Double Adaptive Thresholding method (Supplementary Materials Figure S1). Eventually, thresholds were manually adjusted in some batches to maximize object identification (stem or roots) depending on the image brightness or darkness level. In the third experiment, a defined region in each image (1000 × 1100 pixels) containing the rooting region was used for image segmentation with the GiA Roots software, as described above.

All root system architectural traits and shoot traits measured by GiA Roots were initially selected and were computed directly from the image mask or from the skeleton of the image mask, as described elsewhere [9]. After batch-processing, raw measurements were exported to Excel spreadsheets for data analysis. Other parameters were visually quantified (leaf number, rooting stage, and rooting response) or calculated from previous parameters (e.g., shoot area increase). Measurements of selected traits (Table 1) in the first and second experiment are shown in Supplementary Materials Table S3 for all F_1_ studied lines. In the third experiment, the following additional parameters were also measured: fresh weight and length of stem cuttings measured at planting time (0 dap) and shoot fresh weight, shoot dry weight, shoot water content, shoot growth, root fresh weight, root dry weight, and root water content measured at 40 dap (Table 2 and Supplementary Materials Table S4).

### 4.3. Statistical Analyses

Statistical analyses of the data and descriptors (mean, SD, maximum and minimum, and correlation values) were estimated with StatGraphics Centurion XV software (StatPoint Technologies, United States). Data outliers were identified based on aberrant standard deviation values and were excluded for posterior analyses, as described elsewhere [36]. One-sample Kolmogorov–Smirnov tests were performed to analyze the goodness-of-fit between the distribution of the data and a theoretical normal distribution. Non-parametric tests and data transformation ($y=\frac{1}{x};y=x^{2};y=log_{2}\left(x\right);y=\surd x)$ were applied when needed. To reduce the number of studied traits, we performed multiple correlation tests. To compare the data for a given variable, we performed multiple testing analyses with the ANOVA F-test or the Fisher’s LSD methods. Significant differences were defined as a 5% level of significance (*p*-value < 0.05), unless otherwise indicated. To establish the level of correlation between different morphometric parameters, multiple correlation tests were carried out for selected parameters at different times for all experiments. PCA was performed to check the best-fitting parameter combination in each case at the end of the parameter selection protocol. Star and ray graphs showing differences between selected cultivars in the third experiment were constructed using the Multivariate visualization option and Star and ray chart option in StatGraphics Centurion XV software, comparing average values for all studied traits.

For the statistical analysis of stem cutting ecophysiology traits, nonparametric Kruskall–Wallis tests were assayed. SigmaPlot v12.0 (Systat Software Inc., San José CA, USA) was used for correlation studies and graphical representation.

#### 4.3.1. Estimation of Heterosis in Selfing and Outcrossing F_1_ Populations

Heterosis (H) percentage values were calculated for each studied trait in the first experiment for selfing and outcrossing populations, as described elsewhere [37], and expressed as an increase or decrease of F_1_ hybrid values over mid-parent (relative heterosis, *MP*) or better parent (*BP*) values. Heterosis was calculated as indicated below:

$$H\left(BP\right)=\left(\frac{F1-BP}{BP}\right)x\;100$$

$$H\left(MP\right)=\left(\frac{F1-MP}{MP}\right)x\;100$$

where F1 represents the mean performance of F_1_, BP represents the average better-parent performance, and MP represents the average mid-parental value.

#### 4.3.2. Estimation of Broad-Sense Heritability in Selected Lines

We estimated the broad-sense heritability (*H^2^*) of measured traits in selected lines of the third experiment by means of a multifactorial ANOVA, as described elsewhere [38]. The data values for these selected lines in the first and second experiments allowed us to compare their behaviors in two different environments (e). Heritability was calculated as indicated below:

$$H^{2}=\frac{\sigma^{2}G}{\sigma^{2}G+\left(\frac{\sigma^{2}GE}{e}\right)+\frac{\sigma^{2}E}{re}}$$

where $\sigma^{2}G=\frac{MS_{G}}{re}$; $\sigma^{2}GE=\frac{MS_{GE}-MS_{E}}{r}$; $\sigma^{2}E=MS_{E}$, r represents the number of replicates or individuals per experiment, e represents the number of environments or experiments, MS_G_ represents the mean square of the genotype, MS_GE_ represents the mean square of the interaction between the genotype and the experiment, and MS_E_ represents the mean square error.

### 4.4. Phytohormone Extraction and Analysis

Indole-3-acetic acid (IAA), IAA-aspartate conjugate (IAA-Asp), *trans*-zeatin (*t*Z), salicylic acid (SA), jasmonic acid (JA), abscisic acid (ABA), and the ethylene precursor 1-aminocyclopropane-1-carboxylic acid (ACC) were analyzed in accordance with Reference [39] with some modifications. Cold stored samples were freeze-dried in liquid nitrogen and ground with a pestle into a coarse powder. Powdered samples were homogenized in 0.5 mL of cold (−20 °C) extraction mixture of methanol/water (80/20, *v/v*). Solids were separated by centrifugation (20,000 g, 15 min) and re-extracted for 30 min at 4 °C in an additional 0.5 mL of the same extraction solution. Pooled supernatants were passed through Sep-Pak Plus †C18 cartridges (SepPak Plus, Waters, USA) to remove interfering lipids and part of the plant pigment. They were evaporated at 40 °C under vacuum either to near dryness or until the organic solvent was removed. The residue was dissolved in 1 mL of methanol/water (20/80, *v/v*) solution using an ultrasonic bath. The dissolved samples were filtered through 13 mm diameter Millex filters with nylon membrane (0.22 µm pore size, Millipore, Bedford, MA, USA).

Ten microliters of filtrated extract was injected into a U-HPLC-MS system consisting of an Accela Series U-HPLC (ThermoFisher Scientific, Waltham, MA, USA) coupled to an Exactive mass spectrometer (ThermoFisher Scientific, Waltham, MA, USA) using a heated electrospray ionization (HESI) interface. Mass spectra were obtained using Xcalibur software, version 2.2 (ThermoFisher Scientific, Waltham, MA, USA). For quantification of the plant hormones, calibration curves were constructed for each analyzed component (1, 10, 50, and 100 µg L^−1^) and corrected for 10 µg L^−1^ deuterated internal standards. Recovery percentages ranged between 92% and 95%. The raw data for phytohormone extraction are shown in Supplementary Materials Table S5.

## 5. Conclusions

Vegetative propagation of elite carnation genotypes depends on successful rooting of their stem cuttings. AR formation is a complex developmental process that is strongly influenced by exogenous and endogenous factors. We found a striking variation in rooting performance in a selected F_1_ population derived from a cross between *2003 R 8* and *2101-02 MFR*, which could be used to breed carnation cultivars with enhanced rooting ability to increase production under challenging environmental conditions, such as drought. The leaf water content of the mother plants could be measured in vivo with terahertz spectroscopy technology [40], and this information could be used to predict the adventitious rooting performance of different carnation genotypes. Additional analyses are necessary in order to verify the usefulness of this new technique and its possible implementation in the vegetative mass-production of highly valued ornamental species.

## Figures and Tables

**Table 1:** Selected shoot and root traits measured in the first and second experiments.

Studied Traits	Acronym	Measurement Mode ^a^	Unit
Leaf number	LN_13	Human operator	>1
Average leaf section	ALS_13	Average Width^1^	mm
Cutting height	CH_13	Network Depth^1^	cm
Shoot area	SA_13, SA_20	Network Area^1^	cm^2^
Shoot area increase	ΔSA	SA_20–SA_13	cm^2^
Rooting response	RR_20	Human operator^2^	%
Rooting stage	RS_20	Human operator^2^	0 to 7
Maximum number of roots	MaxNR_20	Maximum number of roots^1^	>1
Average root diameter	ARD_20	Average root width^1^	mm
Root area	RA_20	Network area^1^	cm^2^
Root convex area	RCA_20	Network convex area^1^	cm^2^
Root perimeter	RP_20	Network perimeter^1^	cm

^a^ 1: [9]; 2: [5].

**Table 2:** Selected shoot and root traits measured in the third experiment.

Shoot Traits	Acronym ^a^	Measurement Mode ^b^	Unit
Shoot fresh weight	SFW_0, SFW_40	Precision scale	g
Cutting length	CL_0	Digital caliper	cm
Shoot dry weight	SDW_40	Heater, high precision scale	mg
Shoot water content	SWC	SFW_40 − SDW_40	g
Shoot growth	SG	SFW_40 − SFW_0	g
Root fresh weight/root growth	RFW_40	Precision scale	g
Root dry weight	RDW_40	Heater, high precision scale	mg
Root water content	RWC	RFW_40 − RDW_40	g
Rooting response	RR	Human operator^1^	%
Average root diameter	ARD	Average root width^2^	mm
Root area	RA	Network area^2^	cm^2^
Root convex area	RCA	Network convex area^2^	cm^2^
Root depth	RD	Network depth^2^	cm
Root length	RL	Network length^2^	cm

^a^ Measured at 19, 29, and 40 dap or as otherwise indicated. ^b^ 1: [5]; 2: [9].

## Supplementary Materials

The following are available online at https://www.mdpi.com/2223-7747/8/7/226/s1: Figure S1: Image analysis of carnation stem cuttings; Figure S2: Descriptive traits of the double-cross F1 lines studied in the first experiment; Figure S3: Descriptive traits of the double-cross F1 lines studied in the third experiment; Figure S4: Parameter correlation for different physiological traits in the third experiment; Figure S5: Descriptive traits of the double-cross F1 lines studied in the third experiment. Table S1: Heterosis values for the F1 subpopulations studied; Table S2: Correlation analyses among different physiological traits in the third experiment; Table S3: Raw data for selected parameters in the first and second experiments; Table S4: Raw data for selected parameters in the third experiment; Table S5: Raw data for phytohormones measured.
